# Missing RRI Interpolation Algorithm based on Locally Weighted Partial Least Squares for Precise Heart Rate Variability Analysis

**DOI:** 10.3390/s18113870

**Published:** 2018-11-10

**Authors:** Keisuke Kamata, Koichi Fujiwara, Takafumi Kinoshita, Manabu Kano

**Affiliations:** 1 The Department of Systems Science, Kyoto University, Kyoto 615-8085, Japan; kei.kyoto.be.amb@gmail.com (K.K.); phantom.vib@gmail.com (T.K.); manabu@human.sys.i.kyoto-u.ac.jp (M.K.); 2 The Department of Material Process Engineering, Nagoya University, Nagoya 464-8601, Japan

**Keywords:** R wave detection, heart rate variability analysis, Just-In-Time modeling, locally weighted partial least squares

## Abstract

The R-R interval (RRI) fluctuation in electrocardiogram (ECG) is called heart rate variability (HRV), which reflects activities of the autonomic nervous system (ANS) and has been used for various health monitoring services. Accurate R wave detection is crucial for success in HRV-based health monitoring services; however, ECG artifacts often cause missing R waves and deteriorate the accuracy of HRV analysis. The present work proposes a new missing RRI interpolation technique based on Just-In-Time (JIT) modeling. In the JIT modeling framework, a local regression model is built by weighing samples stored in the database according to the distance from a query and output is estimated only when an estimate is requested. The proposed method builds a local model and estimates missing RRI only when an RRI detection error is detected. Locally weighted partial least squares (LWPLS) is adopted for local model construction. The proposed method is referred to as LWPLS-based RRI interpolation (LWPLS-RI). The performance of the proposed LWPLS-RI was evaluated through its application to RRI data with artificial missing RRIs. We used the MIT-BIH Normal Sinus Rhythm Database for nominal RRI dataset construction. Missing RRIs were artificially introduced and they were interpolated by the proposed LWPLS-RI. In addition, MEAN that replaces the missing RRI by a mean of the past RRI data was compared as a conventional method. The result showed that the proposed LWPLS-RI improved root mean squared error (RMSE) of RRI by about 70% in comparison with MEAN. In addition, the proposed method realized precise HRV analysis. The proposed method will contribute to the realization of precise HRV-based health monitoring services.

## 1. Introduction

The RR interval (RRI) fluctuation in an electrocardiogram (ECG) is known as heart rate variability (HRV), which is a physiological activity reflecting the cardiovascular control exerted by the autonomic nervous systems (ANS) [1,2]. Since the ANS is a control system that acts largely unconsciously and regulates functions such as the heart rate, digestion, respiration, perspiration, and body temperature, it is related to various diseases. Many types of HRV features have been proposed for evaluation of ANS activities, [3,4], and application areas of HRV analysis has been expanded due to recent advances in machine learning technologies. A brief introduction of HRV analysis is described in Appendix A.

It is known that changes in sleep condition affect HRV [5,6]. An HRV-based drowsiness detection method using linear discriminant analysis (LDA) was developed by Vicente et al. [7]. Patel et al. proposed a drowsiness detection algorithm based on a neural network (NN) [8]. A drowsy driving detection algorithm was proposed by integrating HRV analysis and multivariate statistical process control (MSPC), which is a well-known anomaly detection method [9,10].

Apnea affects HRV during sleep because it contributes to the future development of cardiovascular events and HRV reflects the cardiovascular control [11,12]. Sleep apnea can also be screened through monitoring HRV. It has been reported that frequency domain features of HRV are useful for apnea screening [13]. Roche et al. developed a sleep apnea detection method based on HRV features [14].

HRV analysis was also used for epilepsy seizure detection [15], since HRV changes in preictal phases [16,17]. In addition, Fujiwara et al. developed an epileptic seizure prediction algorithm utilizing MSPC, which can predict seizure occurrence prior to onset [18].

Precise long-term RRI measurement devices are needed for realizing these HRV-based health monitoring services. The Holter monitor is used for long-term ECG measurement; however, its use in daily life is difficult since the Holter monitor is expensive and requires operation skills. Yamakawa et al. developed a wearable RRI sensor based on ECG for real-time HRV analysis, which can easily measure RRI [19]. A wearable chemical-electrophysiological sensors for health and fitness monitoring was developed by Imani et al. [20]. In addition, tattoo type sensors for ECG monitoring have been investigated [21,22,23].

**Figure 1:**
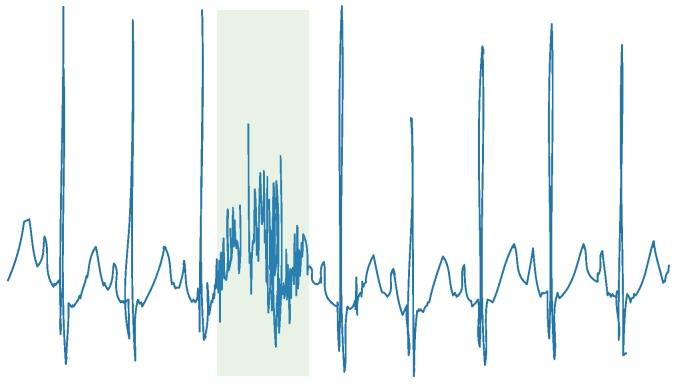
An example of an ECG trace with artifacts (colored band). An R wave cannot be detected due to artifacts.

Adequate HRV feature extraction for RRI data is required. In particular, R wave detection from ECG is essential because HRV feature extraction is easily fluctuated by artifact contamination in the raw RRI data. R waves are not always detected stably due to ECG artifacts caused by body movement or ECG electrode contact failure. Figure 1 illustrates an example of an ECG trace with artifacts, in which the colored band denotes contaminated artifacts.

**Figure 2:**
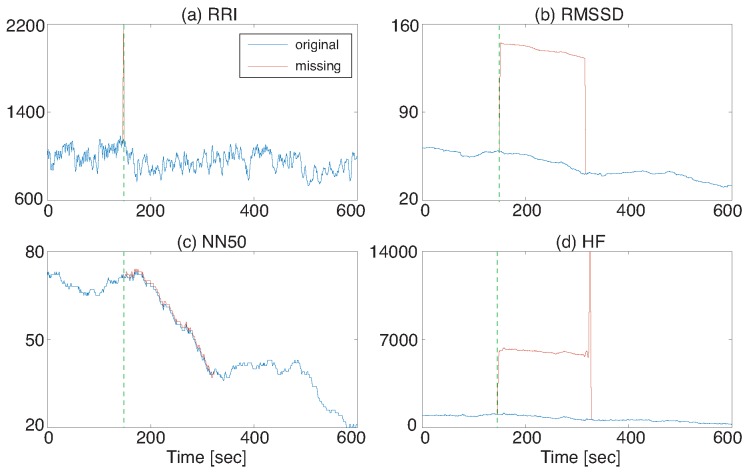
Missing RRI data and its effect on HRV features: (**a**) RRI, (**b**) RMSSD, (**c**) NN50, and (**d**) HF. The HRV features greatly fluctuate when the R detection error occurs.

Some HRV features easily fluctuate when a missing RRI occurs even if it is just one. Figure 2a shows an example of RRI data with one missing and Figure 2b–d are RMSSD, NN50, and HF that were calculated from the RRI data of Figure 2a. A three-minute time window was used for HRV calculation. The vertical line denotes a missing RRI occurrence point. This example shows that some of the extracted HRV features were significantly altered when the R detection error occurred and that their influence lasted for three minutes in this case, which is the window size of HRV calculation.

Although missing R waves may easily be found when the ECG data are analyzed offline, such offline analysis cannot be used for online applications like drowsy driving detection and epileptic seizure prediction.

When the (j+1)th R wave is not detected, $r_{j}$ is as

(1) $$\begin{array}{c} r_{j}={\tilde{r}}_{j}+{\tilde{r}}_{j+1} \end{array}$$

where ${\tilde{r}}_{j}$ is the true RRI measurement when both the *j*th and (j+1)th R waves are detected correctly.

The simplest way of missing RRI treatment is to remove or ignore $r_{j}$ [24]. Although Clifford and Tarassenko recommended using the Lomb-Scargle (LS) periodogram after removing $r_{j}$ for frequency domain feature extraction [25], such treatment cannot be used online because it causes time gaps between real-time. An ectopic RRI modification method from ECG data was proposed by using prior knowledge about arrhythmia [26]. However, its use for missing RRI interpolation is difficult since the effect of R wave detection errors on changes in RRI data is different from that of arrhythmia. Thus, missing RRI should be interpolated appropriately in real-time for precise HRV analysis. Highly adequate interpolation is required particularly in drowsy driving detection and epileptic seizure prediction because their errors caused by missing RRI may lead to severe injuries and accidents.

The electrode contact failure or sensor failure may cause long-term ECG measurement failure. In such cases, HRV analysis and its use for health monitoring should be stopped because we cannot use any information for HRV analysis. Thus, this study focuses on the modification of an isolated R wave detection error.

The present work proposes a new missing RRI interpolation method based on just-in-time (JIT) modeling. JIT modeling or lazy learning is a statistical modeling method that builds a local regression model and that estimates an output using the constructed local model only when an estimate is requested. Although some JIT modeling methods have been proposed [27,28,29,30], the present work adopts locally-weighted partial least squares (LWPLS), which utilizes partial least squares (PLS) for local model construction [31,32]. The proposed method, referred to as LWPLS-based RRI interpolation (LWPLS-RI), interpolates missing RRIs by using a local regression model only when an R wave detection error is detected. R wave detection errors are detected by using a threshold of the measured RRI.

This paper is organized as follows. Section 2 introduces LWPLS and proposes new missing RRI interpolation method. Section 3 validates the performance of the proposed missing RRI interpolation method through a case study of RRI data with artificial missing RRI. Finally, the conclusion and future work are described in Section 4. Although a preliminary version of this work has been reported in [33], the data analyzed in [33] was small, and the performance of the proposed method was not compared with other methodologies.

## 2. Materials and Methods

The present work proposes a new JIT-based algorithm for interpolating missing RRI, which is referred to as LWPLS-based RRI interpolation (LWPLS-RI). This section begins with partial least squares (PLS) and locally-weighted PLS (LWPLS) used in the proposed algorithm.

### 2.1. PLS

PLS is a widely used linear regression method that can build an accurate model with a small number of latent variables. Given an input data matrix $X\in {ℜ}^{N\times M}$ whose *n*th row is the *n*th input sample $x_{n}\in {ℜ}^{M}$ and an output data vector $y\in {ℜ}^{N}$ whose *n*th element is the *n*th output sample $y_{n}\in ℜ$. X and y are mean-centered and appropriately scaled. In PLS, the input X and the output y are broken down as follows:

(2) $$\begin{array}{ccc} X & = & {\mathrm{TP}}^{T}+E \end{array}$$

(3) $$\begin{array}{ccc} y & = & \mathrm{Tb}+f \end{array}$$

where $T\in {ℜ}^{N\times K}$ is the latent variable matrix whose columns are the latent variable $t_{k}\in {ℜ}^{N}$ (k=1,⋯,K), $P\in {ℜ}^{M\times K}$ is the loading matrix of X whose columns are the loading vectors $p_{k}\in {ℜ}^{M}$, and $b={\left[b_{1},\cdots ,b_{K}\right]}^{T}$ is the regression coefficient vector of y. *K* denotes the number of adopted latent variables. $E\in {ℜ}^{N\times M}$ and $f\in {ℜ}^{N}$ are errors.

The nonlinear iterative partial least squares (NIPALS) algorithm can be used to construct a PLS model [34]. Suppose that the first to *k*th latent variables $t_{1},\cdots ,t_{k}$, the loading vectors $p_{1},\cdots ,p_{k}$ and the loading $b_{1},\cdots ,b_{k}$ are given. The (k+1)th residual input and output can be expressed as follows:

(4) $$\begin{array}{ccc} X_{k+1} & = & X_{k}-t_{r}p_{k}^{T}, \end{array}$$

(5) $$\begin{array}{ccc} y_{k+1} & = & y_{k}-b_{k}t_{k}. \end{array}$$

$t_{k}$ is a linear combination of the columns of $X_{k}$, that is, $t_{k}=X_{k}w_{k}$ where $w_{k}\in {ℜ}^{M}$ is the *k*th weighting vector. It is defined so that the covariance between $y_{k}$ and $t_{k}$ is maximized under $\left|\right|w_{k}\left|\right|=1$. Using the Lagrange multipliers method, the function to maximize can be defined as

(6) $$\begin{array}{c} G_{k}=y_{k}^{T}t_{k}-\mu (||w_{k}\left||-1\right)=y_{k}^{T}X_{k}w_{k}-\mu (||w_{k}||-1), \end{array}$$

where μ is the Lagrange multiplier. By solving $\partial G_{k}/\partial w=0$, $w_{k}$ is derived as

(7) $$\begin{array}{c} w_{k}=\frac{X_{k}^{T}y_{k}}{\left|\right|X_{k}^{T}y_{k}\left|\right|}. \end{array}$$

The *k*th loading vector $p_{k}$ and the *k*th loading $b_{k}$ are as follows:

(8) $$\begin{array}{c} p_{k}=\frac{X_{k}^{T}t_{k}}{t_{k}^{T}t_{k}},\;\;b_{k}=\frac{y_{k}^{T}t_{k}}{t_{k}^{T}b_{k}}. \end{array}$$

Finally, the above procedure is repeated until the number of adopted latent variables *K* is achieved; *K* can be determined by cross validation.

Instead of using the Lagrange multipliers method, the derivation of the weighting vectors $w_{k}$ in the NIPALS algorithm can be formulated as an eigenvalue problem. $w_{k}$ is the eigenvector corresponding to the maximum eigenvalue of the following eigenvalue problem:

(9) $$\begin{array}{c} X_{k-1}^{T}y_{k-1}^{T}y_{k-1}X_{k-1}w_{k}=\lambda w_{k}, \end{array}$$

where λ is an eigenvalue.

Algorithm 1 describes the eigenvalue-based NIPALS algorithm for PLS modeling.
**Algorithm 1** NIPALS.1: Set *K*.2: $X_{0}=X-\bar{X}$.3: $y_{0}=y-\bar{y}$.4: **for**k=1 to *K* **do**5:  Derive the eigenvector $w_{k}$ which corresponds to the maximum eigenvalue of the following eigenvalue problem: $X_{k-1}^{T}y_{k-1}^{T}y_{k-1}X_{k-1}w_{k}=\lambda w_{k}$.6:  $t_{k}=X_{k-1}w_{k}$.7:  $p_{k}=X_{k-1}^{T}t_{k}/t_{k}^{T}t_{k}$.8:  $b_{k}=y_{k-1}^{T}t_{k}/t_{k}^{T}t_{k}$.9:  **if** k=K **then**10:   Output $P=[p_{1},\cdots ,p_{K}]$ and $b={\left[b_{1},\cdots b_{K}\right]}^{T}$.11:  **end if**12: **end for**

### 2.2. Locally-Weighted Partial Least Squares

In general, a global linear model cannot function well when a system has strong nonlinearity or changes in characteristics with time. The use of nonlinear modeling methods, such as support vector machine (SVM) or artificial neural network (ANN), is the first choice; however, nonlinear modeling methods are not always applicable because a considerable amount of data is required for nonlinear modeling. Also, it is difficult to even for nonlinear models to cope with changes in system characteristics with time.

Another method for dealing with these problems is JIT modeling, which has the following features:

- Store new samples into a database when available.
- Construct a local model by the samples located in the neighboring region around a query and estimate an output only when estimation is required.
- Discard the constructed local model after its use for output estimation.

In JIT modeling, samples for local modeling should be selected appropriately.

LWPLS is an expansion of PLS based on the framework of JIT modeling for dealing with nonlinearity and system characteristics change. In LWPLS, a local PLS model is built by weighted samples stored in a database according to the similarity between the query and the weighted samples only when an estimate is requested. The constructed local model represents a nonlinear relationship between the input and the output around the query because a nonlinear relationship can be approximated as a linear relationship in a small region. After being used for estimation, the used local model is purged [31,32].

Let X and y have already been stored in a database. When an estimate is requested for a query $x_{q}$, the similarity $\omega_{n}$ between $x_{q}$ and the *n*th sample $x_{n}\left(n=1,\cdots ,N\right)$ is calculated, and a local PLS model is built by the weighted samples with a similarity matrix $\Omega \in {ℜ}^{N\times N}$ defined as:

(10) $$\begin{array}{c} \Omega =\mathrm{diag}[\omega_{1},\omega_{2},\cdots ,\omega_{N}]. \end{array}$$

The similarity between the query $x_{q}$ and a sample $x_{n}$ $\omega_{n}$ in this work is defined as:

(11) $$\begin{array}{ccc} \omega_{n} & = & e^{\left(-d_{n}\varphi /\sigma_{d}\right)}, \end{array}$$

(12) $$\begin{array}{ccc} d_{n} & = & \sqrt{{\left(x_{n}-x_{q}\right)}^{T}\left(x_{n}-x_{q}\right)} \end{array}$$

where $\sigma_{d}$ denotes the standard deviation of $d_{n}\;\left(n=1,2,\cdots ,N\right)$ and φ is a localization parameter; the similarity decreases steeply when φ is small and gradually when φ is large. When the similarity matrix Ω is an identity matrix, LWPLS becomes the original PLS.

Algorithm 2 describes a procedure of LWPLS based on the NIPALS algorithm. Steps 4–7 derive the latent variable t, the loading vector p, and the regression coefficient vector *b* iteratively. In step 6, $w_{k}$ is calculated as the eigenvector of $X_{k}^{T}\Omega y_{k}y_{k}^{T}\Omega X_{k}$, which corresponds to the maximum eigenvalue. The final estimate is output when k=K. A localization parameter φ and the number of latent variables *K* are tuning parameters, which are determined by trial and error or cross-validation.

**Algorithm 2** LWPLS.
1: Set *K* and φ.2: Calculate the similarity matrix Ω3: **for**r=1 to *K* **do**4:  Calculate $X_{k}$, $y_{k}$, and $x_{q,k}$;
(13)$$X_{k}=X-1_{N}\left[\begin{array}{cccc} {\bar{x}}_{1} & {\bar{x}}_{2} & \cdots & {\bar{x}}_{M} \end{array}\right]$$
(14)$$y_{k}=y-1_{N}\bar{y}$$
(15)$$x_{q,k}=x_{q}-{\left[\begin{array}{cccc} {\bar{x}}_{1} & {\bar{x}}_{2} & \cdots & {\bar{x}}_{M} \end{array}\right]}^{T}$$
(16)$${\bar{x}}_{m}=\frac{\sum_{n=1}^{N}\omega_{n}x_{nm}}{\sum_{n=1}^{N}\omega_{n}}$$
(17)$$\bar{y}=\frac{\sum_{n=1}^{N}\omega_{n}y_{n}}{\sum_{n=1}^{N}\omega_{n}}.$$5: Set ${\hat{y}}_{q}=\bar{y}$.6:  Derive the *k*th latent variables of X and y, and the *r*th latent variable of $x_{q}$;
(18)$$\begin{array}{c} t_{k}=X_{k}w_{k},\;t_{q,k}=x_{q,k}^{T}w_{k}. \end{array}$$7:  Derive the *k*th loading vectors of X and the *r*th regression coefficient y;
(19)$$\begin{array}{c} p_{k}=X_{k}^{T}\Omega t_{k}/t_{k}^{T}\Omega t_{k},\;d_{k}=y_{k}^{T}\Omega t_{k}/t_{k}^{T}\Omega t_{k}. \end{array}$$8:  Update ${\hat{y}}_{q}={\hat{y}}_{q}+t_{q,k}d_{k}$.9:  **if** k=K **then**10:   Output ${\hat{y}}_{q}$ as an estimate.11:  **else**12:   Calculate $X_{k+1}$, $y_{k+1}$, and $x_{q,k+1}$;
(20)$$X_{k+1}=X_{k}-t_{k}p_{k}^{T}$$
(21)$$y_{k+1}=y_{k}-t_{k}d_{k}$$
(22)$$x_{q,k+1}=x_{q,k}-t_{q,k}p_{k}.$$13:  **end if**14: 
**end for**



### 2.3. Missing RRI Interpolation

  When the (j+1)th R wave is not detected and the measured *r*th R wave is expressed as Equation (1), only the *j*th RRI estimate ${\hat{r}}_{j}$ is required because the next RRI estimate ${\hat{r}}_{j+1}$ can be calculated from $r_{j}$ and ${\hat{r}}_{j}$: ${\hat{r}}_{j+1}=r_{j}-{\hat{r}}_{j}$. At this time, successive missing occurrences are not considered.

Appropriate input variables should be determined for interpolating missing RRI by LWPLS. Multiple past RRI measurements $r_{j-1},\cdots ,r_{j-L+1}$ and the current measurement $r_{j}$ are used as input variables;

(23) $$\begin{array}{c} x_{j}=\left[r_{j}/2,r_{j-1},\cdots ,r_{j-L}\right] \end{array}$$

where *L* is the number of past measurements. Although the current measurement may be useful for interpolation, the measured $r_{j}$ is about double of ${\tilde{r}}_{j}$. Thus, its average $r_{j}/2$ is used as input.

The procedure of the proposed LWPLS-RI is described in Algorithm 3. Before missing RRI interpolation starts, the initial RRI buffer has to be stored for more than the buffer size *W*. In step 4, $\bar{r}$ is the threshold for finding an R wave detection error. When the RRI measurement $r_{j}$ exceeds $\bar{r}$, it is determined that an R wave detection error has occurred. The threshold $\bar{r}$ is a predetermined parameter to be tuned beforehand; however, the default value of $\bar{r}$ can be set to 1,500 msec. In step 5, the newly measured RRI $r_{j}$ is queued into the RRI buffer in a first-in-first-out (FIFO) manner when an R wave is detected correctly. On the other hand, ${\hat{r}}_{j}$ is estimated by LWPLS when an R wave detection error occurs in steps 8–10. Finally, $r_{j}$ is replaced by the estimated ${\hat{r}}_{j}$ and ${\hat{r}}_{j+1}$ which are queued into the RRI buffer in a FIFO manner in steps 11 and 12. The proposed LWPLS-RI has four tuning parameters: the localization parameter φ, the numbers of latent variables *K* and past RRI measurements used for an input *L*, and the buffer size *W*.

**Algorithm 3** LWPLS-RI.
1: Set ϕ, *R*, $\bar{r}$, and *l*.2: 
**while do**
3:  Measure the *j*th RRI $r_{j}$.4:  **if** $r_{j}\leq \bar{r}$ **then**5:   Enqueue $r_{j}$ into the RRI buffer in the FIFO manner.6:   Wait until the next RRI $r_{j+1}$ is measured.7:  **else**8:   Construct an input $x_{j}$ as Equation (23) from the RRI buffer.9:   Estimate the *j*th RRI ${\hat{r}}_{j}$ from $x_{j}$ by using LWPLS.10:   Calculate the j+1 RRI estimate; ${\hat{r}}_{j+1}=r_{j}-{\hat{r}}_{k}$.11:   Replace $r_{j}$ by ${\hat{r}}_{j}$ and ${\hat{r}}_{j+1}$.12:   Enqueue ${\hat{r}}_{j}$ and ${\hat{r}}_{j+1}$ into the RRI buffer in the FIFO manner.13:   Wait until the next RRI $r_{j+2}$ is measured.14:  **end if**15: 
**end while**



In the proposed algorithm, the RRI data collected from any persons can be used for the initial RRI buffer. Even when the RRI data collected from persons other than users are stored in the initial RRI buffer, the stored RRI data are replaced by the RRI data measured from users themselves through the FIFO manner in steps 11 and 12 in Algorithm 3.

## 3. Results and Discussion

This section evaluates and discusses the interpolation performance of the proposed LWPLS-RI through its application to RRI data in which missing RRIs were introduced artificially. Long-term ECG measurement failure and an ectopic RRI caused by arrhythmia were not considered here.

### 3.1. Simulation Procedure

This case study used the MIT-BIH Normal Sinus Rhythm Database for objective data construction [35]. ECG data measured from subjects 1–18 were clipped from the database and ECG data containing strong artifacts were eliminated. The R waves in the clipped ECG data were detected by using the peak detection algorithm, and each RRI was calculated. Each of eighteen pieces of RRI data, $r^{\left[s\right]}$, was divided into three datasets for parameter optimization of LWPLS $r_{o}^{\left[s\right]}$, initial RRI buffer in Algorithm 3, $r_{b}^{\left[s\right]}$, and validation $r_{v}^{\left[s\right]}$, where s(s=1,⋯,18) denotes the subject index. The numbers of samples in $r_{o}^{\left[s\right]}$, $r_{b}^{\left[s\right]}$, and $r_{v}^{\left[s\right]}$ were 6000, 500, and 5000, respectively. There was no R wave detection error in any of these datasets.

**Figure 3:**
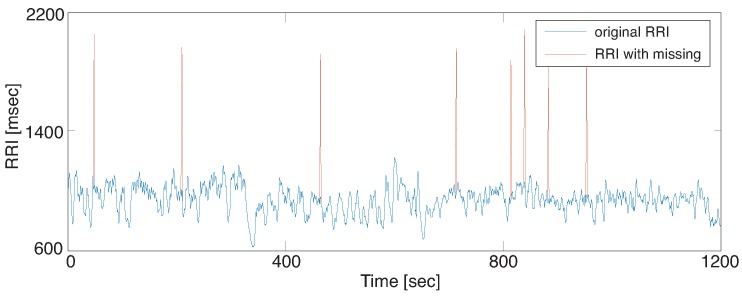
Example of missing RRI with α=0.5%: some RRIs were intentionally eliminated randomly.

Missing RRIs were artificially introduced to the parameter optimization dataset $r_{o}^{\left[s\right]}$ and the validation datasets $r_{v}^{\left[s\right]}$ in a random manner as $r_{j}^{\prime }=r_{j}+r_{j+1}$ and eliminated $r_{j+1}$. There were no successive missing RRIs, and the missing rates were α=0.3%, 0.5%, and 1%. Since the ordinal heart rate of a healthy adult is about 60–80 bpm, 1% missing means that R wave detection error occurs about every 1.5 min. Figure 3 illustrates an example of RRI data with α=0.5 which was generated from the RRI data of subject 16 $r_{v}^{\left[16\right]}$.

**Figure 4:**
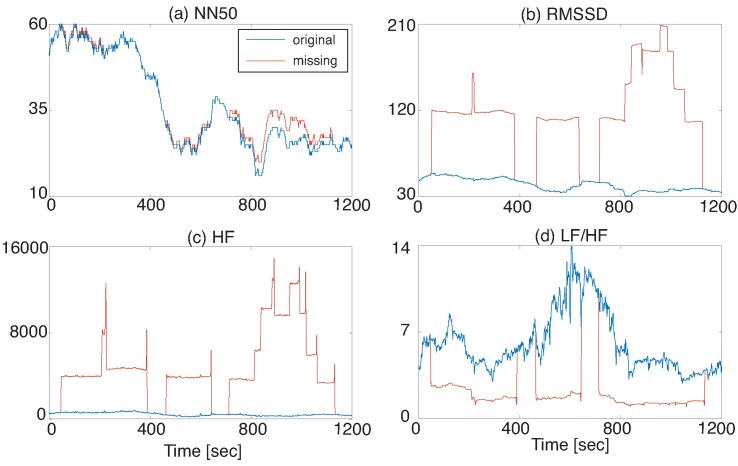
HRV features extracted from the missing RRI: (**a**) NN50, (**b**) RMSSD, (**c**) HF, and (**d**) LF/HF. There were large errors in HRV features due to frequent missing RRI

In this case study, the HRV features described in Appendix A were extracted. A rectangular sliding window whose size is three minutes was used. The time domain features were extracted directly from the raw RRI data. For frequency domain feature extraction, the RRI data were interpolated by the third-order spline and resampled at 4 Hz. An AR model of order 40 was used to calculate frequency domain features. These HRV feature extraction settings were determined based on [18]. Figure 4 shows NN50, RMSSD, HF, and LF/HF extracted from the data in Figure 3. HRV features except for NN50 change greatly due to missing RRIs and such a situation may deteriorate the performance of HRV-based monitoring services.

Before applying the proposed LWPLS-RI to the validation datasets $r_{v}^{\left[s\right]}$, appropriate parameters in LWPLS were determined using the parameter optimization datasets $r_{o}^{\left[s\right]}$. 1% of R wave detection errors artificially occurred in all $r_{o}^{\left[s\right]}$ in a random manner. The localization parameter φ and the number of latent variables *K* in LWPLS were determined so that the root mean squared error (RMSE) between the true and interpolated RRIs was minimized. Here, the RRI buffer size *W* and the number of past RRIs used for input *L* were fixed to 500 and 3. The determined parameters were φ=1.3 and K=3, respectively.

The proposed method with the determined parameters was applied to the validation datasets $r_{v}^{\left[s\right]}\;\left(s=1,\cdots ,18\right)$. Although step 4 in Algorithm 3 judges missing RRI occurrences, missing RRI positions were known in this case study for interpolation performance evaluation. The following three interpolation methods were tested for comparison:

- MEAN: Replace the missing RRI $r_{j}$ by a mean of $x_{j}$.
- Equal Division (ED): Replace $r_{j}$ by the value of $r_{j}/\left(q+1\right)$, where *q* is the number of successive R wave detection errors.
- PLS-RI: Replace $r_{j}$ by an output of a PLS model whose input is $x_{j}$.

The ectopic RRI remove method [24] was not tested in this case study because it can not be used in online applications. After interpolation by these methods, RMSE between the true and interpolated RRIs and RMSE between HRV features derived from the true and interpolated RRIs were calculated.

In this case study, missing RRI generation in the validation datasets $r_{v}^{\left[k\right]}$ and missing RRI interpolation were repeated 30 times for precise performance evaluation.

### 3.2. Interpolation Results

**Figure 5:**
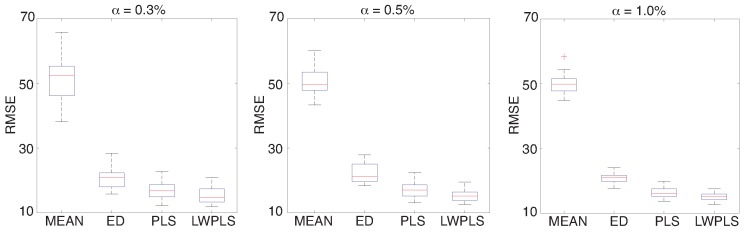
RMSE of RRI interpolated by MEAN, ED, PLS, and LWPLS when α=0.3% (**left**), 0.5% (**center**), and 1% (**right**) when a simulation was repeated 30 times using all of the eighteen pieces of validation data. The proposed LWPLS-RI achieved the best interpolation performance.

Figure 5 shows application results of four missing RRI interpolation methods to RRI data with α=0.5% in Figure 3 and HRV extraction from the interpolated RRI data. These RMSEs of the four methods were calculated through a simulation repeated 30 times using all of the eighteen pieces of validation data. RMSEs of PLS-RI and the proposed LWPLS-RI were lower than those of MEAN and ED, and LWPLS-RI was the best. The proposed LWPLS-RI improved RMSE about 70% in comparison with MEAN when α=0.5%. In addition, the interpolation performance hardly changed even if the error rate increased.

**Figure 6:**
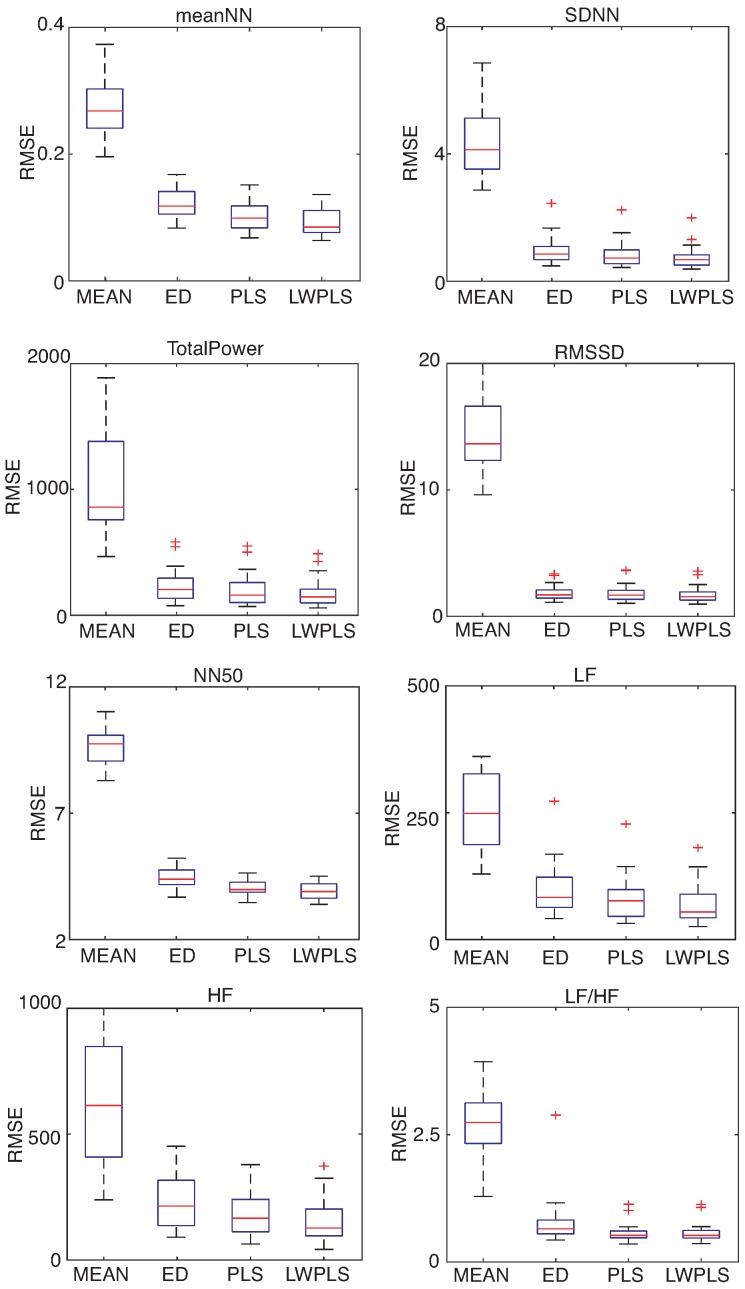
RMSE of HRV features derived from RRIs interpolated by MEAN, ED, PLS, and LWPLS when α=0.3%. These eight HRV features were explained in the Appendix A. RMSEs of the four methods were calculated through a simulation repeated 30 times using all of the eighteen pieces. The proposed LWPLS-RI achieved the best HRV analysis performance.

**Figure 7:**
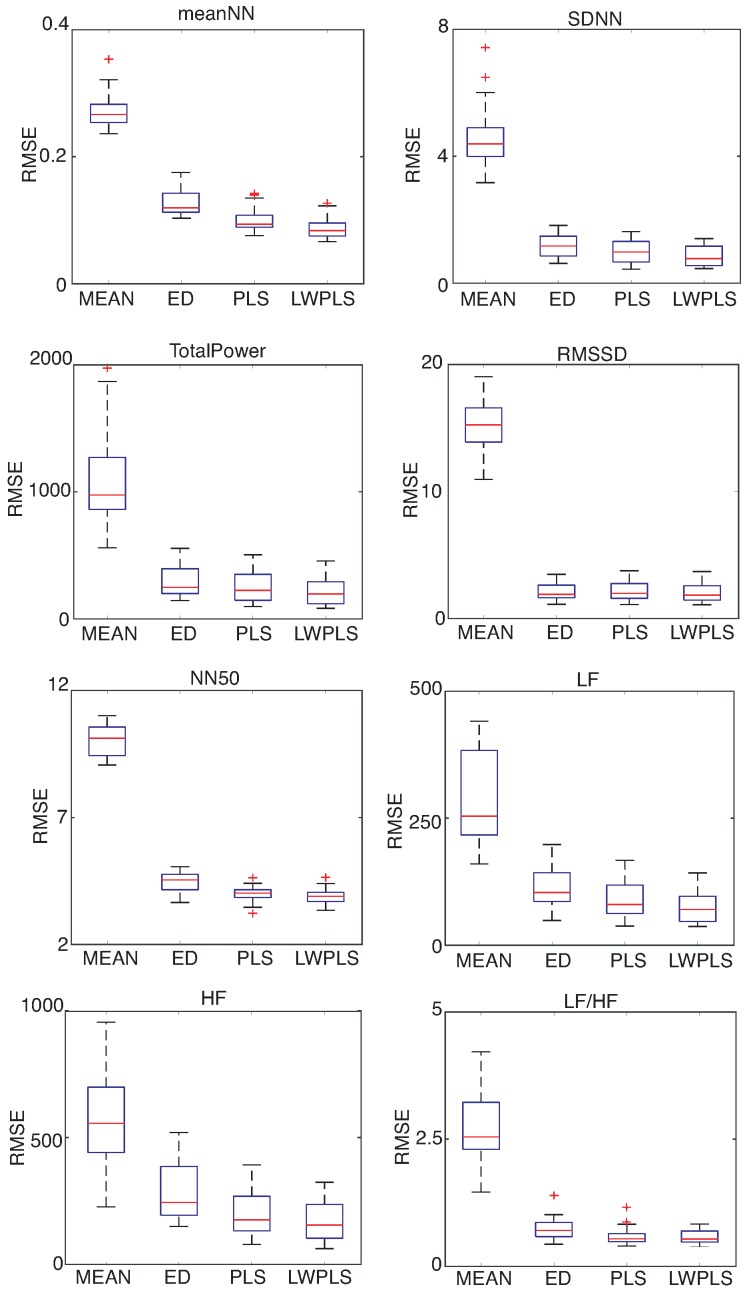
RMSE of HRV features derived from RRIs interpolated by MEAN, ED, PLS, and LWPLS when α=0.5%. These eight HRV features were explained in the Appendix A. RMSEs of the four methods were calculated through a simulation repeated 30 times using all of the eighteen pieces. The proposed LWPLS-RI achieved the best HRV analysis performance.

**Figure 8:**
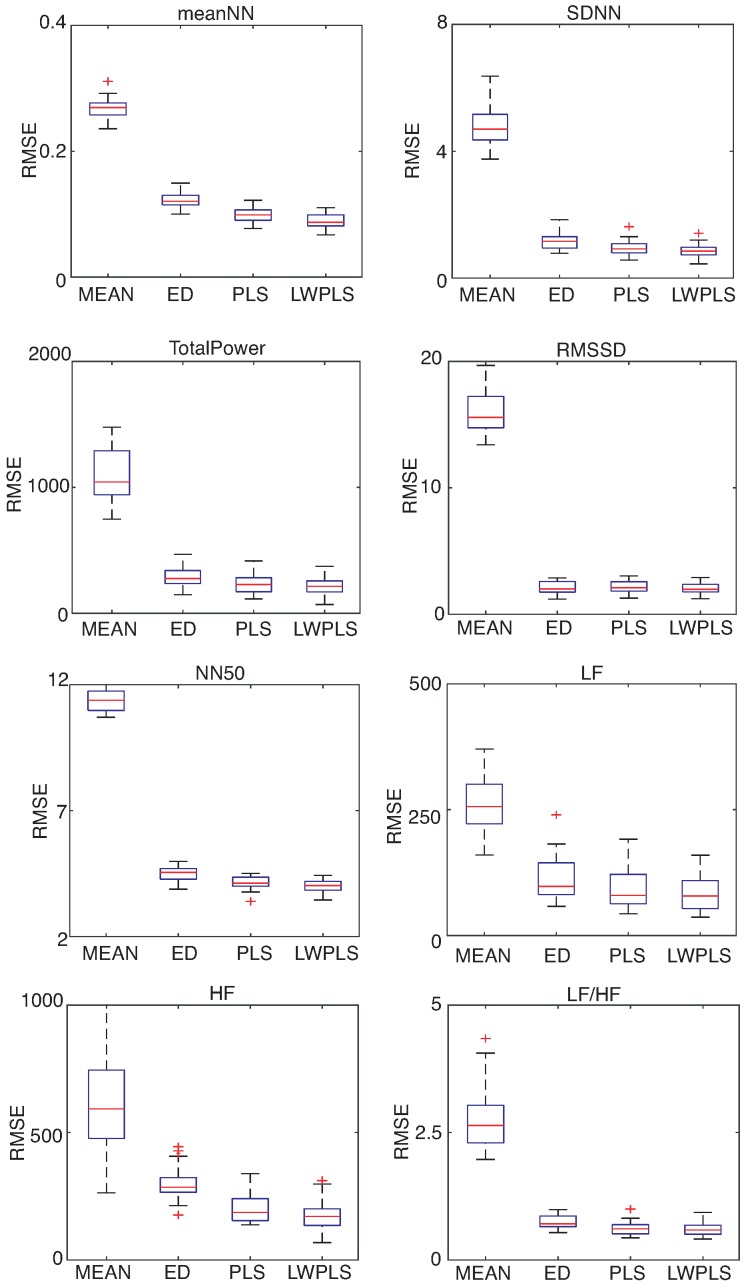
RMSE of HRV features derived from RRIs interpolated by MEAN, ED, PLS, and LWPLS when α=1.0%. These eight HRV features were explained in the Appendix A. RMSEs of the four methods were calculated through a simulation repeated 30 times using all of the eighteen pieces. The proposed LWPLS-RI achieved the best HRV analysis performance.

HRV features were derived from the RRI data interpolated by four methods. Figure 6, Figure 7 and Figure 8 show RMSEs of eight HRV features calculated by the interpolated RRIs through a simulation repeated 30 times. The proposed LWPLS-RI achieved the highest performance.

These results demonstrate the usefulness of the proposed LWPLS-RI for HRV analysis from RRI data with missing RRIs.

### 3.3. Discussion

**Figure 9:**
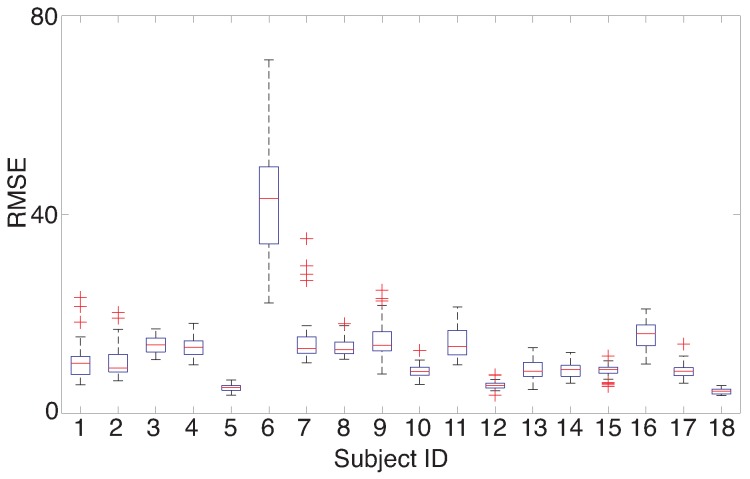
RMSE of RRI interpolation in each subject by LWPLS when α=0.5%. Although RMSEs of almost all subjects except Subject 6 were less than twenty, that of Subject 6 was more than forty.

According to the simulation, the proposed LWPLS-RI achieved the best performance of missing RRI interpolation, of which the mean of RMSEs calculated from all subjects was 15.1 when the error rate was α=1%. However, there were differences among subjects as shown in Figure 9. RMSE calculated from subject 6 was worse than the other subjects. None of the interpolation methods could improve his/her RMSE. The standard deviations of the raw RRI data of subject 6 and subject 5 whose RMSE was small were 61.8 and 140.4, respectively, which clearly showing that RRI fluctuation of subject 6 was much larger than that of subject 5 although there were no RRI detection errors. This indicates that a missing RRI cannot be interpolated appropriately when the fluctuation range of RRI is huge. LWPLS uses the past information most similar to a sample to be estimated; however, the number of past similar RRIs for interpolation becomes small when RRIs fluctuate largely.

The current RRI measurement $r_{j}\left(={\tilde{r}}_{j}+{\tilde{r}}_{j+1}\right)$ is used for utilizing missing information. To investigate the effect of using the current measurement, RRI interpolation without $r_{j}$ was tested. The number of past RRIs used for input *L* was L=4 so that the total number of input variables did not change. As a result, the means of RMSE by MEAN, PLS-RI, and LWPLS-RI were 49.1, 34.2, and 31.9, respectively, which are worse than RRI interpolation with $r_{j}$. This shows that the current RRI measurement $r_{j}$ should be used for interpolation in any method.

In the proposed LWPLS-RI, the RRI buffer size *W* and the number of past RRIs used for input *L* were fixed to 500 and 3 in this case study. To investigate the effect of these parameters on the interpolation performance of the proposed LWPLS-RI, different parameter settings were compared. The RRI buffer size *W* was changed to W=100,300,500, and 1000 and other parameters were fixed to the same values as Section 3.1. RMSEs were calculated using validation data with α=0.5% in a simulation repeated 30 times. The means of RMSE were 15.9, 15.4, 15.7, and 15.1 when W=100,300,500, and 1000, respectively. These numbers show that the interpolation performance was not improved when a large buffer size was selected, and in fact became worse when W=100. Thus, W=300 is sufficient for RRI interpolation.

The number of past RRIs used for input *L* was changed to L=2,3,4, and 5, and other parameters were fixed to the same values as Section 3.1, except the number of latent variables *K* which was determined using the parameter optimization dataset for every different *L*. RMSEs were calculated using validation data with α=0.5% in a simulation repeated 30 times. The means of RMSE were 12.7, 12.8, 12.9, and 12.8 when L=2,3,4, and 5, which shows that the interpolation performance did not change, regardless of which *L* was selected.

In addition, the proposed LWPLS-RI was applied to the RRI data containing two successive missing RRIs and compared with the single RRI missing case. In this simulation, two successive missing RRIs were randomly generated at five different points in each validation dataset, and they were interpolated sequentially by LWPLS-RI whose parameters were the same as Section 3.1. The following input was used for the interpolation instead of Equation (23):

(24) $$\begin{array}{c} x_{j,1}=\left[r_{j}/3,r_{j-1},\cdots ,r_{j-L}\right]. \end{array}$$

The interpolated RRI for the first missing ${\hat{r}}_{j,1}$ was added to the input for the second interpolation as

(25) $$\begin{array}{c} x_{j,2}=\left[\left(r_{j}-{\hat{r}}_{j,1}\right)/2,{\hat{r}}_{j,1},r_{j-1},\cdots ,r_{j-L+1}\right]. \end{array}$$

Using these inputs, the RRI data containing two successive missing RRIs were interpolated by LWPLS-RI sequentially, and RMSEs of the first and the second interpolations were calculated. This procedure was repeated 30 times. The mean of RMSE of the first missing interpolation was 24.9, which was worse than that of the single missing interpolation. More information about RRI was lost in the successive missing case than the single missing case, which makes it difficult to interpolate RRI. Thus, it becomes more difficult to interpolate missing RRIs when more than three successive missing RRIs occur. However, the mean of RMSE of the second missing interpolation was 15.2 which is almost the same as the single missing interpolation case. This may have been caused by input variable construction for the second interpolation. Equation (25) uses the interpolated first RRI ${\hat{r}}_{j,1}$. Information lost through successive missing RRIs was recovered to some extent by the first interpolation and ${\hat{r}}_{j,1}$ contained such recovered information that may be useful for the second missing interpolation.

The proposed LWPLS-RI can be easily implemented in mobile computers such as a smartphone because the computational load is much lighter than methods that need to process ECG signals directly. This is important from the viewpoint of practical use. An HRV-based epileptic seizure prediction smartphone app has already been developed and tested in hospitals [18]. In addition, an HRV-based drowsy driving detection smartphone app has been commercialized [9]. The performances of these smartphone apps will be improved through appropriate RRI interpolation by the proposed method.

It is concluded that the proposed LWPLS-RI has the potential for realizing highly accurate HRV-based health monitoring services in the future.

## 4. Conclusions

A missing RRI interpolation method was proposed utilizing the framework of JIT modeling, whereby a local model is constructed, and missing RRI is estimated by the constructed model only when R wave detection errors occur. The result of applying the proposed LWPLS-RI to real RRI data with artificial missing RRIs showed that it interpolated missing RRI more appropriately than other methods. The proposed method is not applicable to long-term ECG measurement failure since it modifies only one RRI. HRV analysis should be stopped during long-term ECG measurement failure.

The limitations of this study include the properties of the objective data collected from the Physionet database, such as the limited number of subjects and the fact that all subjects were healthy and did not have arrhythmia or other cardiovascular diseases.

In the future work, we will develop a unified framework for interpolating and modifying ectopic RRI data caused by arrhythmia as well as R wave detection errors.

## Appendix A Heart Rate Variability Analysis

This appendix introduces HRV analysis. A typical ECG trace consists of some peaks, and the highest peak is called an R wave. The RR interval (RRI) [ms] is defined as an interval between an R wave and the next R wave. The R wave is detected by using a derivative-based peak detection algorithm [36]. Every RRI changes due to ANS activities, and this phenomenon is called HRV. Standard HRV features are classified into time domain features and frequency domain features [1].

The time domain features are obtained from the original RRI data.

- meanNN: Mean of RRI.
- SDNN: Standard deviation of RRI.
- Total Power (TP): Variance of RRI.
- RMSSD: Root means square of the difference of adjacent RRI.
- NN50: Number of pairs of adjacent RRI, whose difference is more than 50 ms.

Frequency domain features are defined based on the power spectrum density (PSD) of the resampled RRI data, which is derived by Fourier analysis or an autoregressive (AR) model.

- LF: Power of the low-frequency band (0.04–0.15Hz) in PSD. LF reflects the activity of both the sympathetic and parasympathetic nervous systems.
- HF: Power of the high-frequency band (0.15–0.4Hz) in PSD. HF reflects the parasympathetic nervous system activity.
- LF/HF: Ratio of LF to HF. LF/HF expresses the balance between the sympathetic nervous system activity and the parasympathetic nervous system activity.

For frequency analysis, the raw RRI data are interpolated by using spline and are resampled at equal intervals, because they are not sampled at equal intervals. Appropriate settings of HRV analysis should be determined depending on applications; however, the HRV analysis guideline recommends that RRI data should be measured for more than two minutes and that the sampling rate should be at least 200 Hz [1].
